# Treatment of pediatric intracranial aneurysms: institutional case series and systematic literature review

**DOI:** 10.1007/s00381-024-06384-x

**Published:** 2024-04-18

**Authors:** Michael G. Brandel, Jillian H. Plonsker, Robert C. Rennert, Gautam Produturi, Megana Saripella, Arvin R. Wali, Carson McCann, Vijay M. Ravindra, David R. Santiago-Dieppa, J. Scott Pannell, Jeffrey A. Steinberg, Alexander A. Khalessi, Michael L. Levy

**Affiliations:** 1 Department of Neurosurgery, University of California, San Diego-Rady Children’s Hospital, San Diego, CA USA; 2 Department of Neurosurgery, University of Utah, 175 North Medical Drive East, Salt Lake City, CA USA; 3 School of Medicine, University of California, San Diego, CA USA

**Keywords:** Cerebral aneurysm, Outcomes, Clipping, Endovascular, Coiling, Flow diversion

## Abstract

**Introduction:**

Pediatric intracranial aneurysms (IAs) are rare and have distinct clinical profiles compared to adult IAs. They differ in location, size, morphology, presentation, and treatment strategies. We present our experience with pediatric IAs over an 18-year period using surgical and endovascular treatments and review the literature to identify commonalities in epidemiology, treatment, and outcomes.

**Methods:**

We identified all patients < 20 years old who underwent treatment for IAs at our institution between 2005 and 2020. Medical records and imaging were examined for demographic, clinical, and operative data. A systematic review was performed to identify studies reporting primary outcomes of surgical and endovascular treatment of pediatric IAs. Demographic information, aneurysm characteristics, treatment strategies, and outcomes were collected.

**Results:**

Thirty-three patients underwent treatment for 37 aneurysms over 18 years. The mean age was 11.4 years, ranging from one month to 19 years. There were 21 males (63.6%) and 12 females (36.4%), yielding a male: female ratio of 1.75:1. Twenty-six (70.3%) aneurysms arose from the anterior circulation and 11 (29.7%) arose from the posterior circulation. Aneurysmal rupture occurred in 19 (57.5%) patients, of which 8 (24.2%) were categorized as Hunt-Hess grades IV or V. Aneurysm recurrence or rerupture occurred in five (15.2%) patients, and 5 patients (15.2%) died due to sequelae of their aneurysms. Twenty-one patients (63.6%) had a good outcome (modified Rankin Scale score 0–2) on last follow up. The systematic literature review yielded 48 studies which included 1,482 total aneurysms (611 with endovascular treatment; 656 treated surgically; 215 treated conservatively). Mean aneurysm recurrence rates in the literature were 12.7% and 3.9% for endovascular and surgical treatment, respectively.

**Conclusions:**

Our study provides data on the natural history and longitudinal outcomes for children treated for IAs at a single institution, in addition to our treatment strategies for various aneurysmal morphologies. Despite the high proportion of patients presenting with rupture, good functional outcomes can be achieved for most patients.

## Introduction

Pediatric intracranial aneurysms (IA) are exceedingly rare lesions with potentially devastating outcomes. While they account for only 1–7% of all IAs, their unique morphologies, locations, and treatment strategies render them distinct from adult IAs [16, 27, 31]. Numerous case series have described the heterogeneity in pediatric IA morphology, with saccular, fusiform, and dissecting subtypes among the most commonly reported [14, 41]. Although some childhood IAs arise in the setting of preexisting conditions, multisystem disease, or trauma, many arise without provocation or known risk factors, leading to numerous theories regarding their pathogenesis [16]. 

Treatment strategies include both open surgical and endovascular techniques, and depend on the aneurysm size, type, location, patient age, physician/institutional preference and comorbidities [14, 29, 36]. Open surgical techniques such as clipping have long been the standard for aneurysm treatment, but endovascular techniques such as coiling, stent-assisted coiling, and flow diversion have become increasingly used for both adult and pediatric patients [1, 30]. Endovascular approaches have been associated with favorable short-term outcomes; however, endovascular treatment may carry an increased risk of aneurysm recurrence, an important consideration for children [31]. 

Long-term outcomes vary, and depend on the initial presentation, aneurysm location and treatment method. A meta-analysis by Yasin et al. reported an overall favorable outcome rate (modified Rankin score of 0, 1, or 2) of 85%. Mortality rates range from 5 to 28%, with the majority occurring because of rupture, and a Hunt & Hess grade of IV or V conferring markedly worse outcomes [1, 10, 12, 16, 27, 41]. 

We report our 18-year experience with pediatric IAs and the evolving approach to care at our institution. We also review the current literature regarding the management of these rare lesions and provide a foundation for future work.

## Methods

### Study design

We retrospectively reviewed the electronic medical record for patients age ≤ 20 years who were treated for IAs between 2003 and 2020 at Rady Children’s Hospital/University of California, San Diego. IAs associated with arteriovenous malformations (AVMs) were excluded from the study. IAs were typically detected with noninvasive imaging initially due to patient symptomatology or as incidental findings. Digital subtraction angiography (DSA) was used for further characterization when indicated. Patients with subarachnoid hemorrhage (SAH) were classified according to the Hunt & Hess scale, and their CT scans were scored using the Fisher scale. Aneurysm size was classified as small (≤ 10 mm), large (11–25 mm), or giant (≥ 25 mm). Patients were offered endovascular or open surgical treatment depending on IA characteristics and clinical condition. Data regarding perioperative complications, recurrence, retreatment, and functional outcomes were recorded. Patients were followed up at frequent intervals with surveillance imaging.

### Data analysis

R v4.2.2, RStudio (The R Foundation), and the “tidyverse” package were used for statistical analysis [37, 39]. α was set at 0.05. Univariate comparisons between open and endovascular treatment groups were completed using independent-sample t test or Welch’s unpaired t-test for continuous variables, and chi-square analysis or Fisher’s exact test for categorical variables. Outcomes of interest included mortality and modified Rankin score at last follow-up. For survival analysis, patients were followed from the time of presentation until the time of their death or the point of last contact. Univariate evaluation of potential prognostic variables was performed with Kaplan-Meier curves and univariate Cox regression analysis.

### Systematic literature review

A systematic review of the literature from 1991 to 2022 was performed using the PubMed, Embase, and Web of Science databases. Search terms included (“ped” OR “pipeline” OR “flow diverter” OR “flow diversion” OR “endovascular” OR “coil” OR “coiling” “OR “open surgery” OR “clip” OR “clipping”) AND “aneurysm” AND (“pediatric” OR “paediatric” OR “children” OR “child”). Prospective and retrospective studies were included if they presented primary clinical data and outcomes for pediatric IAs. Secondary data from meta-analyses or literature reviews were excluded. Studies were also excluded if they were classified as abstracts, editorials, expert opinions, or letters. Duplicate articles and data were removed. Data from included studies were collected to summarize the current pediatric IA literature. This work is in accordance with the PRISMA (Preferred Reporting Items for Systematic Review and Meta-Analyses) guidelines.

## Results

### Patient characteristics

From 2003 to 2020, 33 patients were treated for 37 aneurysms (Table 1). The mean age was 11.4 years. There were 21 males and 12 females (64% vs. 36%), a male predominance of 1.75:1. Most patients were Hispanic (17, 51.5%), with the remainder being White (10, 30.3%), Asian (5, 15.2%), or Black (1, 3.0%). Aneurysmal rupture was present at initial presentation in 19 (57.6%) patients. Of patients who presented with rupture, 11 (57.9%) had Hunt & Hess grades of IV or V SAH. An external ventricular drain (EVD) was placed in 14 (42.4%) patients.

**Table 1:** Pediatric aneurysms treated at Rady Children’s Hospital between 2005 and 2020

Patient Number	Age (years)	Sex	Presentation	Vessel	Size	Morphology	Treatment	Obliteration	Outcome (last follow-up mRS)
					(mm)				
1	8	M	Headache	L ICA		Fusiform	Clipping	Yes	0
2	0.5	M	SAH IV	R AChA	7.5	Saccular	Clipping	Yes	4
3	9	M	SAH III	R AComm		Saccular	Clipping	Yes	0
4	14	M	Traumatic ICH	L PICA		Ovoid	Clipping	Yes	4
5	5	M	ICH, seizure	R pericallosal a.		Saccular	Clipping	Yes	6
6	17	M	Traumatic postoperative aneurysm resulting in visual deficit	R ICA		Fusiform	Clipping and wrapping	Yes	0
7	10	F	SAH III	L PCA		Mycotic	Clipping	Yes	0
8	4	F	SAH IV	R MCA	7.8	Saccular	Clipping	Yes	1
9	14	M	Traumatic GSW	R ICA		Dissecting	Coiling then ligation	Yes	0
10	0.1	F	ICH	L MCA	3	Tumor-associated	Clipping	Yes	6
11	16	M	SAH II	R ICA	9	Blister	Clipping	Yes	1
12	12	F	SAH V, IVH	Basilar tip		Fusiform	Clipping and resection	Yes	6
13	0.1	M	IVH, IPH, thrombosed aneurysm	Unknown, likely R anterior circulation		Thrombosed	Resection and lobectomy	Yes	3
14	13	M	SAH V, IPH	R PComm	15	Saccular	Clipping	Yes	1
15	1	M	Incidental	R MCA	14	Saccular	Clipping	Yes	0
16	15	F	SAH II	L ICA	10	Saccular	Clipping, then flow diversion	Yes	0
17	7	F	SAH V	R PCA	34	Pseudoaneurysm	Coiling, then resection	Yes	2
18	4	M	Headache, seizure	R MCA		Fusiform, thrombosed	Clipping	Yes	0
19	12	M	Headache, thrombosed aneurysm	L VA	4	Fusiform, dissecting	Coiling	Yes	1
20	17	M	MCA stroke	L MCA		Saccular, partially thrombosed	Clipping	Yes	0
21	17	F	SAH V	L ICA		Saccular, multilobulated	Clipping	Yes	4
22	12	F	SAH III	L AComm (2x), L PComm	3	Saccular	Clipping (2x Acomm); clipping, then coiling (PComm)	Yes	1
23	0.1	F	SAH I, ICH	R PICA	8	Saccular	PICA-PICA bypass	Yes	3
24	10	F	Headache	L VA	26	Complex	Coiling and vessel sacrifice	Yes	0
25	11	M	Incidental	L MCA	9	Saccular	Clipping	Yes	0
26	19	F	Incidental	L ICA (2x)		Dumbbell	PED (both aneurysms)	Yes	0
27	2	M	Developmental delay	L VA	36	Saccular	Clipping	Yes	2
28	15	M	Incidental	L ACA		Pseudoaneurysm	Clipping	Yes	2
29	14	M	SAH V	L VA	7.5	Complex	Clipping	Yes	3
30	17	F	Aneurysm rupture, ICH	R MCA	25	Fusiform	PED	No	6
31	1	M	TIA	R MCA (2x)		Multiple dissecting	Stent-assisted coiling, then coil sacrifice	Yes	6
32	5	M	Aneurysm rupture, ICH	R MCA		Fusiform	Clipping	Yes	6
33	14	M	Headache, nausea, vomiting	L ICA		Complex, fusiform, multilobulated	PED, then balloon angioplasty	Yes	1

SAH graded according to Hunt & Hess classification. SAH = subarachnoid hemorrhage. IPH = intraparenchymal hemorrhage. IVH = intraventricular hemorrhage. ICH = intracranial hemorrhage. TIA = transient ischemic attack. GSW = gunshot wound. ICA = internal carotid artery. ACA = anterior cerebral artery. MCA = middle cerebral artery. PCA = posterior cerebral artery. VA = vertebral artery. AChA = anterior choroidal artery. AComm = anterior communicating artery. PComm = posterior communicating artery. PICA = posterior inferior cerebellar artery. PED = pipeline embolization device

### Aneurysm features: location, size, and morphology

Of the 37 aneurysms treated, 26 (70.3%) arose from the anterior circulation, and 11 (29.7%) arose from the posterior circulation; 19 (51.4%) of these aneurysms were located on the left side, and 17 (46%) were on the right, with one (3.0%) aneurysm arising in the midline from the basilar artery. The most common aneurysm morphologies were saccular (*n* = 13, 39%) and fusiform (*n* = 8, 24%), with the remainder (*n* = 16, 43.2%) being pseudoaneurysmal (*n* = 2, 6.1%), complex (*n* = 2, 6.1%), dissecting (*n* = 2, 6.1%), blister (*n* = 1, 3.0%), dumbbell (*n* = 1, 3.0%), mycotic (*n* = 1, 3.0%), ovoid (*n* = 1, 3.0%), thrombosed (*n* = 1, 3.0%), and tumor-associated (*n* = 1, 3.0%). The median aneurysm size was 10 millimeters (IQR [8, 26]), with 17 (52, four (12%) large aneurysms, and 12 (36%) giant aneurysms (Table 2).

**Table 2:** Patient demographics and clinical characteristics

Characteristic	Overall *n = 33 patients*	Endovascular *n = 8 patients*	Open *n = 25 patients*	*p*-value^2^
Age, median (IQR) *n = 33 patients*	11.4 (4.0, 13.8)	13.1 (9.1, 14.7)	10.4 (3.8, 13.6)	0.2
Sex, n (%) *n = 33 patients*				0.4
F	12 (36.4%)	4 (50.0%)	8 (32.0%)	
M	21 (63.6%)	4 (50.0%)	17 (68.0%)	
Ethnicity, n (%) *n = 33 patients*				0.3
Asian	5 (15.2%)	2 (25.0%)	3 (12.0%)	
Black	1 (3.0%)	1 (12.5%)	0 (0.0%)	
Hispanic	17 (51.5%)	3 (37.5%)	14 (56.0%)	
White	10 (30.3%)	2 (25.0%)	8 (32.0%)	
Primary aneurysm laterality, n (%) *n = 33 patients*				> 0.9
Left	16 (48.5%)	4 (50%)	12 (48.0%)	
Midline	1 (3.0%)	0	1 (4%)	
Right	16 (48.5%)	4 (50%)	12 (48.0%)	
Primary aneurysm vessel, n (%) *n = 33 patients*				> 0.9
Internal carotid a.	8 (24.2%)	3 (37.5%)	5 (20.0%)	
Anterior cerebral a.	1 (3.0%)	0	1 (4.0%)	
Middle cerebral a.	9 (27.3%)	2 (25.0%)	7 (28.0%)	
Posterior cerebral a.	2 (6.1%)	1 (12.5%)	1 (4.0%)	
Basilar a.	1 (3.0%)	0	1 (4.0%)	
Vertebral a.	4 (12.1%)	2 (25.0%)	2 (8.0%)	
Anterior choroidal a.	1 (3.0%)	0	1 (4.0%)	
Anterior communicating a.	1 (3.0%)	0	1 (4.0%)	
Posterior communicating a.	1 (3.0%)	0	1 (4.0%)	
Posterior inferior communicating a.	2 (6.1%)	0	2 (8.0%)	
Pericallosal a.	1 (3.0%)	0	1 (4.0%)	
Other/unknown vessels	2 (6.0%)	0	2 (8.0%)	
Primary aneurysm morphology, n (%) *n = 33 patients*				0.014
Saccular	13 (39.4%)	0	13 (48.0%)	
Fusiform	8 (24.2%)	3 (37.5%)	5 (20%)	
Other	12 (36.4%)	5 (62.5%)	7 (28.0%)	
Patients with large or giant aneurysms, n (%) *n = 33 patients*	16 (48.5%)	4 (50.0%)	12 (48.0%)	> 0.9
Initial treatment, n (%) *n = 33 patients*				N/A
Clip ligation	23 (69.7%)	23 (92.0%)	-	
Resection/lobectomy	1 (3.0%)	1 (4.0%)	-	
Vascular bypass	1 (3.0%)	1 (4.0%)	-	
Coil embolization	4 (12.1%)	-	4 (50.0%)	
Pipeline stenting	3 (9.1%)	-	3 (37.5%)	
Stent-assisted coil embolization	1 (3.0%)	-	1 (12.5%)	
Modified Rankin score, n (%) *n = 33 patients*				0.8
0	12 (36.4%)	3 (37.5%)	9 (36.0%)	
1	6 (18.2%)	2 (25.0%)	4 (16.0%)	
2	3 (9.1%)	1 (12.5%)	2 (8.0%)	
3	3 (9.1%)	0 (0.0%)	3 (12.0%)	
4	3 (9.1%)	0 (0.0%)	3 (12.0%)	
6	6 (18.2%)	2 (25.0%)	4 (16.0%)	
Ruptured Status, n (%) *n = 33 patients*				0.2
Ruptured	19 (57.6%)	3 (37.5%)	16 (64%)	
Unruptured	14 (42.4%)	5 (62.5%)	9 (36%)	
Aneurysm size n (%) *n = 33 patients*				0.6
Small	17 (51.5%)	5 (62.5%)	12 (48%)	
Large/giant	16 (48.5%)	3 (37.5%)	13 (52%)	
Anterior v posterior n (%) *n = 33 patients*				0.1
Anterior	23 (69.7%)	7 (87.5%)	16 (64%)	
Posterior	10 (30.3%)	1 (12.5%)	9 (36%)	
EVD placement prior to surgery n (%) *n = 33 patients*				0.8
Yes	14 (42.4%)	3 (37.5%)	11 (44%)	
No	19 (57.8%)	5 (62.5%)	14 (56%)	

### Treatments and outcomes

25 patients (75.8%) underwent open surgery and eight (24.2%) underwent endovascular treatment as their initial intervention; of these patients, 23 (92%) had clip ligation. Initial clip ligation was accompanied by open resection, hematoma evacuation, and aneurysmal wrapping in one (4%) patient each. Finally, IPH evacuation followed by PICA-PICA bypass and open resection followed by lobectomy was performed in one (4%) patient each. Within the endovascular group, three (37.5%) patients had coil embolization, three (37.5%) had pipeline embolization device (PED) stent placements and one (12.5%) patient each had stent-assisted coil embolization and coil embolization with vessel sacrifice. Ten (30.3%) patients required more than one surgery, of which three (30%) were unplanned (one repeat clipping due to aneurysmal recurrence, one coil-assisted vessel sacrifice due to aneurysm re-rupture after stent-assisted coiling, and one balloon angioplasty due to an endoleak after placement of a PED). Two (6.1%) patients required a third intervention to adequately treat their lesions. Recurrence of a treated lesion occurred in one (3.0%) patient (originally treated via clip ligation), and frank re-rupture occurred in four (12.1%) patients (two clip ligations, one PED, and one stent-assisted coil embolization), with two of these events resulting in patient death.

Four (12.1%) patients had complications because of their interventions. All patients had undergone open intervention (although one complication was secondary to intraoperative angiogram). Intraoperative rupture occurred for one patient undergoing clip ligation and resection of a fusiform basilar tip aneurysm, resulting in death 17 days postoperatively. One patient with a giant right saccular AChA aneurysm developed an intracranial abscess after clipping which required surgical evacuation. Another patient with a small right saccular MCA aneurysm developed a CSF leak after clipping which required repair. Finally, one patient who underwent clipping of a giant left vertebral artery saccular aneurysm suffered a left femoral artery thrombus after intraoperative endovascular access and required anticoagulation.

Postoperatively, there were six (18.2%) patients with new deficits, including visual field impairment, vocal cord paralysis, and extremity/hemibody weakness. Deficits were temporary in four (12.1%) patients and permanent in two (6.0%) patients. Six (15.7%) required a permanent shunt, all of whom had undergone open surgery, three of whom had previously required EVD placement, and five of whom had initially presented with aneurysm rupture. At the time of latest follow-up, 12 (36.4%) patients had a modified Rankin score (mRs) of 0, six (18.2%) had a score of 1, three (9.1%) had a score of 2, three (9.1%) had a score of 3, four (9.1%) had a score of 4, and six (18.2%) patients were deceased (Table 2).

#### Survival analysis

**Fig. 1:**
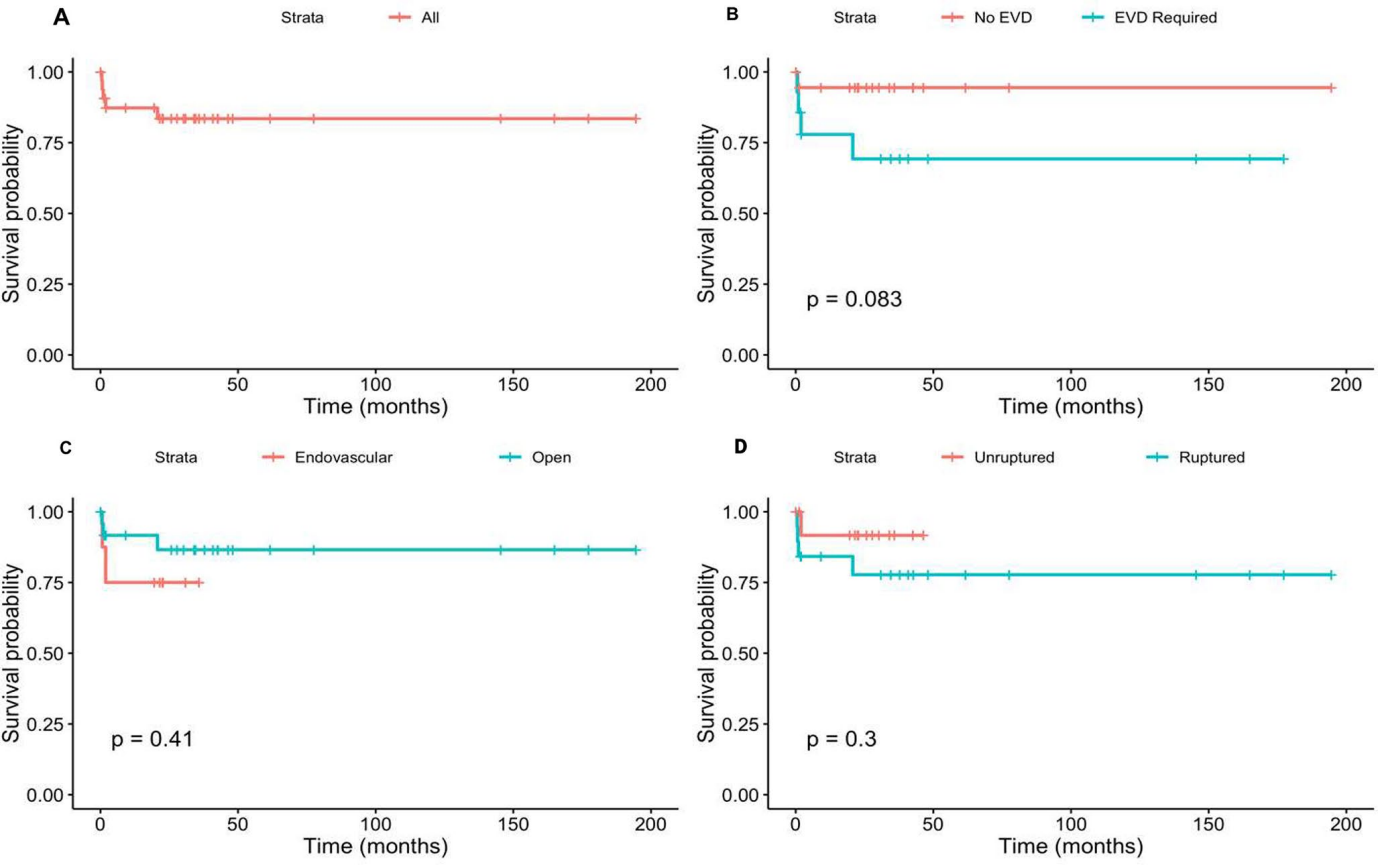
**A**) Kaplan-Meier curve assessing mortality in all patients. **B**) Kaplan-Meier curve comparing mortality in patients who did or did not require an external ventricular drain (EVD). **C**) Kaplan-Meier curve comparing mortality in patients treated with endovascular versus open surgical methods. **D**) Kaplan-Meier curve comparing mortality in patients with ruptured and unruptured aneurysms

Age, sex, aneurysm circulation (anterior vs. posterior), size, rupture status, EVD placement, and treatment modality (open vs. endovascular) were investigated using univariate chi-square tests, t-tests, Kaplan Meier survival analysis (Fig. 1). There were no factors significantly associated with mortality (Table 3). Overall, 1-year survival was 89% and 5-year survival was 78%. Univariate Cox regression survival analysis did not reveal any significant prognostic factors affecting mortality (Table 4).

**Table 3:** Outcome analysis by subgroup

Characteristic	Unfavorable outcome *n = 12*	Favorable outcome *n = 21*	*p*-value
Age			0.7
0–12 years	8.0 (66.7%)	12.0 (57.1%)	
13–18 years	4.0 (33.3%)	9.0 (42.9%)	
Sex			0.7
Female	5.0 (41.7%)	7.0 (33.3%)	
Male	7.0 (58.3%)	14.0 (66.7%)	
Aneurysm size			0.9
Small	6.0 (50.0%)	11.0 (52.4%)	
Large/giant	6.0 (50.0%)	10.0 (47.6%)	
Ruptured Status			0.003
Unruptured	1.0 (8.3%)	13.0 (61.9%)	
Ruptured	11.0 (91.7%)	8.0 (38.1%)	
Primary aneurysm location			> 0.9
Anterior circulation	8.0 (66.7%)	15.0 (71.4%)	
Posterior circulation	4.0 (33.3%)	6.0 (28.6%)	
EVD placement prior to surgery			0.004
No	3.0 (25.0%)	16.0 (76.2%)	
Yes	9.0 (75.0%)	5.0 (23.8%)	
Hunt-Hess Score			0.2
1–3	8.0 (72.7%)	3.0 (37.5%)	
4–5	3.0 (27.3%)	5.0 (62.5%)	
Initial treatment modality			0.7
Endovascular	2.0 (16.7%)	6.0 (28.6%)	
Open	10.0 (83.3%)	15.0 (71.4%)	

**Table 4:** Univariate Cox regression analysis

Variables	Coefficient	Standard Error	*p*-value	HR	95% CI
Age	-0.034	0.073	0.64	0.97	0.22–0.64
Sex	-0.11	0.92	0.90	0.89	0.15–5.4
Anterior vs. Posterior	-0.50	1.1	0.65	0.61	0.068–5.4
Size Classification	-0.25	0.91	0.78	0.78	0.13–4.6
Rupture Status	1.1	1.1	0.33	3	0.33–27
EVD Placement	1.7	1.1	0.21	5.6	0.62–50
Open vs. Endovascular	-0.73	0.91	0.43	0.48	0.43–15

### Systematic literature review

**Fig. 2:**
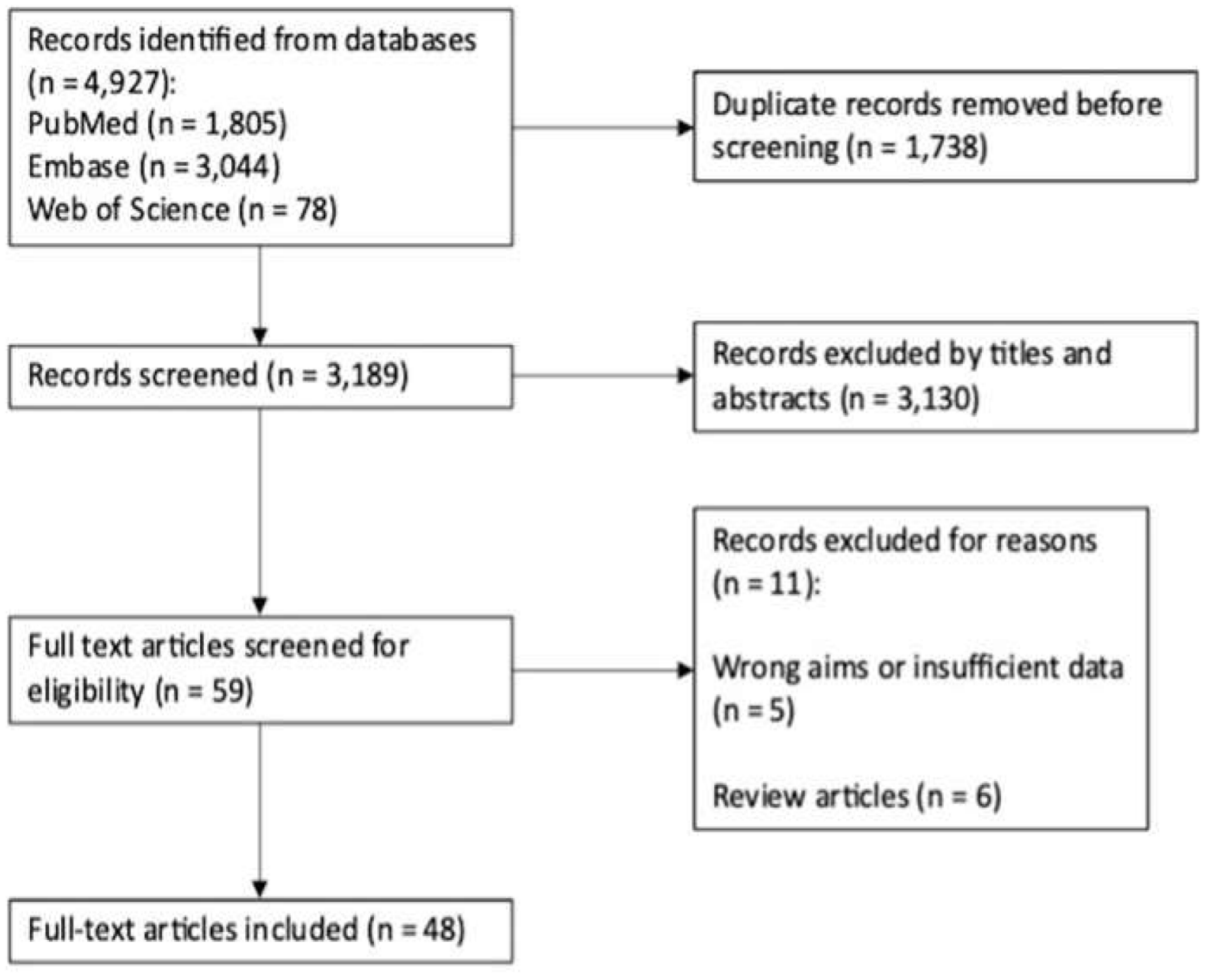
Flow diagram of literature search and selection of included studies

The literature search across the databases yielded 4,927 publications, of which 48 were included in our literature review summary (Fig. 2). In total, these encompassed 1482 aneurysms (mean of 31 aneurysms per study). There were 611 endovascular and 656 microsurgical procedures. The average age was 11 years old with a mean follow-up time of 3.9 years. Unweighted mean rates of aneurysm recurrence were 12.7% for those treated endovascularly and 3.9% treated microsurgically.

## Discussion

While pediatric IAs share some characteristics with their adult counterparts, decades of observation and treatment have established them as distinct entities, defined by unique risk factors, treatment strategies, and prognoses. In this work, we report our experience with pediatric IAs over an 18-year period and compare our results to previously published case series and cohort studies identified in our systematic literature review. All patients were managed by the senior author in collaboration with the endovascular neurosurgery service, building on his experience treating 70 aneurysms in patients < 20 years of age prior to 2003.

### Epidemiological features

Numerous studies have established the unique epidemiological and clinical characteristics of pediatric IAs. Most commonly reported is a predilection for males, with ratios in the literature ranging from 1.2:1 to 3:1 [12, 21, 22, 32]. Similarly, our cohort had a male predominance of 1.75. Several authors have also described a bimodal age distribution, in which IAs commonly become symptomatic predominantly by mass effect or SAH within the first two years of life, and again in late adolescence [3, 34]. We did not observe this pattern, although this may be due in part to the inherent rarity of these lesions as well as our limited sample size.

### Aneurysm characteristics

While no single parent artery was clearly predominant, the MCA and ICA were the most represented within our cohort, with 12 and 9 (28.5% and 21.4%) IAs arising from these arteries, respectively. Mehrotra et al. reported MCA/ICA incidence rates of 24.7% & 15.1% respectively; figures which are similar to those by Yasin et al., Garg et al., and others [3, 8, 12, 41]. 

Children lack many of the risk factors traditionally associated with aneurysm formation in adults, such as hypertension, smoking, and age, and thus many are thought be idiopathic [16]. There may however be a genetic component, as these lesions have been linked with several inherited diseases, such as polycystic kidney disease, aortic coarctation, and sickle cell anemia [26]. Vasculopathies such as Marfan syndrome and Ehlers-Danlos syndrome are implicated in approximately 10% of all pediatric IAs [7, 19]. Pediatric IAs have also been known to affect the posterior circulation at a higher rate than their adult counterparts. While reports vary, most estimates fall between 10% and 40%, consistent with our observed rate of 26.1%, compared to approximately 6% in adults [3, 6, 30]. 

Pediatric IAs are also structurally distinct. Saccular aneurysms, which encompassed over 90% of adult cases, account for only 30–70% in children, and represented 38.1% of aneurysms in our cohort [9, 11]. Together with fusiform aneurysms (16.7%), these classically described morphologies only comprised just over half of the observed variants in our cohort. This morphological heterogeneity is noted in the literature, many of which report ratios of atypical aneurysms ranging from 30 to 50% [8, 21, 34, 41]. To account for these unique morphologies, Lasjaunias et al. utilized a novel classification system that combined physical architecture with clinical context to classify aneurysms as saccular, fusiform, infectious (mycotic), or traumatic [15, 21]. IAs in children tend to be quite large, with giant aneurysms comprising 3–37% of IAs reported in published case series and 34.3% in our study [21, 25, 31, 33]. 

### Presentation and radiological findings

Children with IAs commonly present with headache, ranging from 50 to 90% in the literature including patients with SAH, and 50% in our cohort [4, 12, 16]. Headaches are multifactorial, and may be caused by mass effect of large or giant aneurysms, turbulent blood flow, or SAH / hydrocephalus. We had a larger proportion of patients presenting in poor neurological condition (Hunt & Hess grade IV or V) (52.1%, 12/23 patients) than observed in other case series (0–43%) [3, 12, 16, 20, 22, 27, 31, 33, 37, 41]. This is possibly due to our institution’s status as a tertiary and quaternary referral center, routinely caring for patients from Mexico and Guam in addition to Southern California.

### Treatment methods

Recent years have seen the proliferation of various endovascular techniques to treat aneurysms, including primary coiling, balloon or stent-assisted coiling, and flow diversion. Thus far, these minimally invasive methods show comparable outcomes to traditional open microsurgery for both ruptured and unruptured aneurysms, as reported by numerous meta-analyses and case series [1, 2, 13, 32, 41]. Open treatment via clipping seems to minimize the risk of recurrence, while endovascular intervention is less prone to immediate complications, and may be more suitable for more higher-risk patients with more comorbidities [2, 17, 18, 23, 25]. However, many institutions and individual surgeons/interventionalists prefer one approach, making robust comparisons amongst the literature difficult [35, 40]. In our case series, the majority of aneurysms were treated via an open approach, while the remainder were treated with endovascular methods such as coiling or flow diversion.

Flow-diverting stents are relatively recent additions to the armament of the endovascular neurosurgeon, with emerging literature on their use and long-term efficacy in the pediatric population. Navarro et al. first reported the use of flow diverters in three children to good effect, and later work by Vargas et al., among others, established that these devices, at least within the established follow-up periods, were safe and effective methods of treating complex aneurysms in children [4, 9, 34, 38]. An additional important consideration for the long-term stability and efficacy of PEDs and stents in pediatric patients is ensuring appropriate antiplatelet therapy. While these protocols have been researched extensively in adults, there is little information regarding their applicability to children. In their study assessing flow diverters in children, Barburoglu et al. utilized weight-based dosing extrapolated from adult scales for older, larger children, and modified fractional dosing for younger or lower-weight children [4]. The utilization of flow diverters is also limited in the setting of SAH, which is present at diagnosis for many children, as dual antiplatelets are needed which can be associated with higher rates of hemorrhagic complications, as well as the delayed timeframe of aneurysm thrombosis [24]. However, flow diverters are increasingly being used for adult patients in the setting of rupture, particularly for IAs not amenable to other strategies such as blister aneurysms and may warrant exploration for children as well.

### Institutional approach

At our institution, open surgery is the preferred treatment modality for pediatric IAs requiring intervention with amenable morphology. The higher risk of recurrence after endovascular intervention is an important factor for children who may have many decades to live. As shown in our case series, saccular IAs were exclusively clipped while IAs with unusual morphologies and aneurysmal malformations were considered for open or endovascular intervention on a case-by-case basis. Large or thrombosed IAs with mass effect, or ruptured IAs with associated intraparenchymal hemorrhages, were preferentially clipped or resected, while fusiform or dissecting lesions were better treated with endovascular approaches. Specifically, we consider flow diversion for irregularly shaped (e.g. dumbbell, fusiform) IAs that are not amenable to deconstructive techniques (i.e., with coils) due to vessel anatomy.

We frequently use DSA as a diagnostic adjunct, even for IAs considered amenable to open treatment. However, DSA is not without risk in the pediatric patient as the reported complication rates range from 0.4 to 6.7% [5, 7]. The majority of complications seen in these studies, however, were transient neurological deficits, with major complications occurring in 3/587 patients and 1/241 patients, respectively [5, 7]. We observed one DSA-related complication, a femoral artery thrombus requiring anticoagulation in a 2-year-old after intraoperative DSA during craniotomy.

As stent-assisted coiling and flow diversion require dual antiplatelet therapy (DAPT), they are rarely utilized in the setting of rupture to avoid hemorrhagic complications. Optimal antiplatelet regimens are not well established in children, particularly in those age less than 10 [8, 28]. We typically use aspirin plus a Plavix elixir for at least 6 months in those who require DAPT. Children are assessed with serial platelet reactivity tests to determine the minimum weight-based dose to achieve therapeutic effect.

Patients undergo post-intervention surveillance with MRA every 6–12 months to minimize tests involving radiation and undergo follow-up DSA if there is a concern for recurrence.

### Outcomes

We did not identify any factors significantly impacting survival, possibly due to the high survival rate even among ruptured aneurysm patients in our series of 84%. Within the literature, post-treatment mortality rates range from 2 to 35% [14, 15, 16, 20]. Rebleeding from either de novo or recurrent aneurysms is one of the major factors of perioperative mortality in these patients, which underlines the need for consistent follow-up and surveillance imaging [2]. Recurrence occurred in five (13%) patients in our case series; only one recurrence was noted on surveillance imaging, and the other four presented as delayed post-treatment ruptures in which prior follow-up DSAs had demonstrated obliteration. Amelot et al. noted that rebleeding of IAs were associated with a higher mortality rate compared to unruptured aneurysms in their multivariate analysis (OR = 9.2; *p* = 0.02) [2]. Yasin et al. and others have also demonstrated an association between SAH severity (as measured by Hunt & Hess grade) and poor functional status and mortality by measure of Glasgow Outcomes Scale [36, 41]. 

In our series, four (10.5%) patients suffered periprocedural complications; results which are within the range reported in the literature (range 0–54%) [10, 17, 25]. Commonly reported complications in prior studies include intraoperative rupture and bleeding, thrombotic events, transient cranial nerve palsies, and neurocognitive deficits [15, 16, 20]. Recurrent aneurysms or reoperation occurs in a small but significant number of patients. In a study of 51 surgically treated pediatric IA patients, 60% experienced rebleeding, and 23% experienced asymptomatic aneurysm recurrence [2]. Another series of 57 aneurysms found favorable outcomes in the vast majority of cases using endovascular techniques, with complications including three new neurological deficits, two aneurysms recurrences that required retreatment, and one aneurysm rerupture that required retreatment [41]. Regardless of the treatment modality, close follow up is needed as re-treatment may be indicated for aneurysmal neck remnants detected on post-treatment angiograms, de-novo aneurysms that form in patients with connective tissue disorders, or after subsequent (potentially occult) bleeding events.

### Limitations

Limitations of our study include its retrospective design, rarity of the pathology, and the long inclusion period (18 years) during which endovascular technologies have expanded and evolved. Treatment algorithms for rare pathologies such as pediatric IAs rely heavily on resources available at the institution, as well as the treating team’s clinical experience, and thus might not be generalizable to other hospitals. We also acknowledge that the relatively small sample size may have limited power to detect statistical differences between groups.

## Conclusion

We describe our institutional approach to the preoperative evaluation, treatment selection, and postoperative care for a variety of aneurysmal morphologies in children. Intraoperative and postoperative complications were rare, with only one perioperative mortality. We performed a systematic literature review which contextualizes our findings with the existing literature. Our series builds on prior data demonstrating that good functional outcomes can be achieved for most pediatric IA patients.

## Data Availability

No datasets were generated or analysed during the current study.
